# Case Report: Endovascular Treatment of Chronic Atherosclerotic Renal Artery Total Occlusions with Failed Medical Therapy

**DOI:** 10.3389/fsurg.2022.843568

**Published:** 2022-10-18

**Authors:** Pengyu Li, Guochen Niu, Ziguang Yan, Bihui Zhang, Min Yang

**Affiliations:** Department of Interventional Radiology and Vascular Surgery, Peking University First Hospital, Beijing, China

**Keywords:** renal artery occlusion (RAO), endovascular treatment, medical therapy, split renal function, blood pressure

## Abstract

**Background:**

Current guidelines generally no longer support revascularization for chronic renal artery occlusive diseases because results from randomized controlled trials favor medical therapy over angioplasty. However, increasing reports indicate that patients with renal artery occlusion (RAO) can benefit from revascularization under certain circumstances.

**Case summary:**

Here, we present a patient with renal artery stenosis (RAS) who does not have refractory hypertension or fit any clinical trial inclusion criteria by far. Medical therapy failed to prevent the progression of RAS in this patient, leading to total occlusion of his right renal artery. This patient had progressive renal insufficiency but recovered renal function after endovascular treatment.

**Conclusion:**

This case demonstrates that angioplasty can be beneficial in selected RAO patients, especially those with residual renal function and collateral perfusion.

## Introduction

Renal artery stenosis (RAS) is a leading cause of refractory hypertension and ischemic nephropathy with a progressive decline in renal function (1). A principal cause of the occlusive renovascular disease is atherosclerosis, which accounts for nearly 90% of RAS cases (2). Renal artery occlusion (RAO) is defined as occlusion without an anterograde flow of the renal artery, and if a RAO patient presents with hypertension or renal insufficiency for more than 3 months, the occlusion is considered chronic (3). Treatment for RAO lacks guidelines on account of low incidence. The effect of revascularization in RAS aiming at preventing the progression of chronic kidney disease and lowering blood pressure (BP) is controversial compared with medical therapy alone. Herein, we report a case of a 61-year-old patient in whom medications failed to prevent total occlusion of his right renal artery, resulting in progressive renal insufficiency. His renal function recovered after endovascular revascularization and remained stable during a long-term follow-up of 4 years.

## Case Presentation

A 61-year-old male patient came to our hospital for kidney dysfunction. Elevated serum creatinine (142 µmol/L) was found during a routine annual physical examination 3 months before admission. He had been diagnosed with hypertension 20 years earlier and maintained with an angiotensin-converting enzyme inhibitor and calcium channel blocker with good BP control. This patient also had a stroke 4 years earlier with no sequela left. After the stroke, he was treated with daily aspirin 100 mg and atorvastatin 20 mg.

Upon admission to our institution, his BP was 122/74 mmHg with the administration of irbesartan 150 mg and nifedipine controlled-release tablets 30 mg per day. Urinalysis showed no abnormality, and low-density lipoprotein-cholesterol was 1.64 mmol/L. His serum creatinine (Scr) was 133 µmol/L, and the estimated glomerular filtration rate (eGFR) calculated by the modification of diet in renal disease (MDRD) formula corrected for Chinese (4) was 50.65  mL/min/1.73 m^2^. Doppler ultrasound demonstrated severe stenosis in the right renal artery (>70%, RI = 0.47), while the contralateral renal artery was widely patent. The overall length of the right kidney had decreased to 9.6 cm, while the contralateral kidney was 11.3 cm. Split kidney function [split GFR (sGFR)] was then assessed by ^99m^Tc-DTPA (diethylene-triamine-pentaacetate) renal dynamic scintigraphy, and the sGFR of the ischemic kidney was significantly lower than the contralateral kidney (14 mL/min vs. 41 mL/min, respectively).

**Figure 1:**
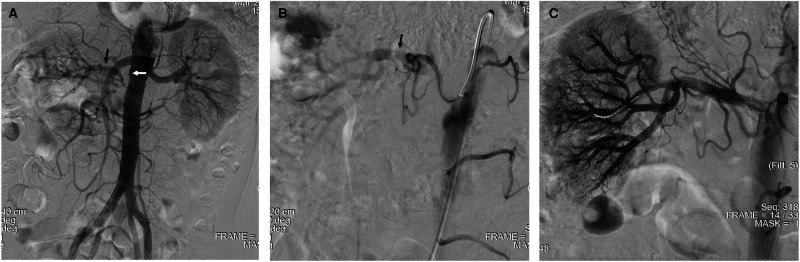
Stenting for occlusive right renal artery. (**A,B**) Angiography demonstrated complete occlusion of the right renal artery with collateral perfusion. (**C**) Ischemic kidney was revascularized after deployment of balloon-expandable stents. The black arrow indicates collateral flow, and the white arrow indicates the occlusive right renal artery.

Angiography showed total occlusion of the right renal artery at the ostial level with collateral perfusion to the right kidney (**Figure 1A,B**). Sequential dilation by balloons up to 4 × 40 mm was performed followed by deployment of balloon-expandable stents and successfully revascularized the ischemic kidney (**Figure 1C**).

**Figure 2:**
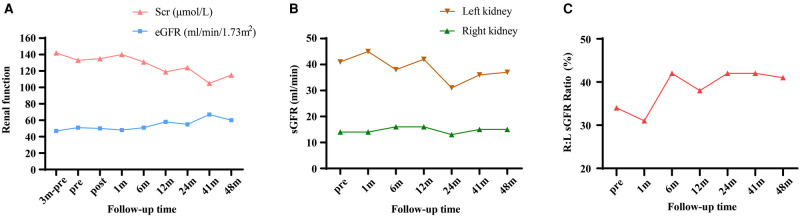
Recovery of renal function after stenting. (**A**) Change of serum creatinine and estimated glomerular filtration rate (GFR). (**B**) Change of GFR in the split kidney. (**C**) Split GFR ratio of the right-to-left kidney. 3m-pre indicates 3 months before treatment, pre indicates pretreatment, the post indicates post-treatment.

This patient continued the same anti-hypertensive therapy and other medications after endovascular treatment. During a 4-year follow-up, his BP has been kept in good control with an average of 130/80 mmHg. Doppler ultrasound observed no sign of restenosis and that the size of the ischemic kidney remained unchanged at the latest follow-up. Scr did not decrease but seemed slightly rebound 1 month after intervention (**Figure 2A**). While during the long-term follow-up, his Scr fell down to the normal range along with a mild recovery of eGFR (**Figure 2A**). The function of the ischemic kidney remained stable, and a rise in the right-to-left sGFR ratio was presented (**Figure 2B,C**).

## Discussion

Revascularization for RAS is controversial. Recent randomized controlled trials (RCTs) have failed to prove any advantages of angioplasty over optimal medical therapy (OMT) alone in RAS patients with respect to either BP control or renal protection (1, 5, 6). Although these trials have been plagued by serious methodological flaws in study design, including inclusion and exclusion criteria and endpoints (2, 7), the latest european society of cardiology/european society for vascular surgery (ESC/ESVC) guidelines (2017) still indicated that routine revascularization is not recommended in RAS secondary to atherosclerosis based on results from these RCTs (8). Indications of revascularization in chronic RAO are more strictly controlled. SCAI Appropriate Use Criteria (AUC) for renal artery stenting (2014) pointed out that it would rarely be appropriate for a treatment to be performed on an occlusive renal artery (2). However, SCAI statement (2017) regarding renal intervention in RAS stuck to the most current american heart association/american college of cardiology (ACC/AHA) guidelines, suggesting the AUC including resistant hypertension, ischemic nephropathy, and cardiac disturbances (9, 10). How to identify proper RAO patients who might benefit from revascularization has been a major concern for vascular specialists. The sudden onset of hypertension or deterioration of BP seemed to be the most common complaint of RAO patients in few published studies, and some patients had refractory hypertension (11, 12). Nonetheless, this patient’s BP had been kept in good control by two kinds of drugs. Some studies suggested that in patients with chronic kidney disease and severe RAS, angioplasty is most beneficial in those with a more rapid decline of renal function (13). However, serum creatinine elevation in this patient was not so sharp compared with previous cases and had been seemingly stable during the past 3 months before hospitalization. We have to admit that it is a patient who does not fit any clinical trial inclusion criteria by far. However, for this patient, comprehensive medications failed to prevent the progression of RAS, resulting in total occlusion; thus, renal artery stenting was performed. Considering that the average progressive GFR decline in RAO patients was 4.1 mL/min per year (14), the validity of revascularization in this patient was reasonable. Patients with symptomatic RAS are reasonable candidates for revascularization when optimal medical therapy fails (15). This case also challenged the efficiency of medical therapy in preventing the progression of RAS.

Most studies just assessed global renal function and do not evaluate split renal function in the ischemic and the contralateral kidney. However, as the reduction in sGFR in the ischemic kidney due to hemodynamically significant RAS may be compensated by hyperfiltration in the contralateral kidney that is exposed to hypertension, total eGFR poorly reflects the progression of the disease and the effects of revascularization (16, 17). Thus, we evaluated split renal function by renal dynamic scintigraphy. Previous studies reported that renal angioplasty would likely increase GFR in stenotic kidneys while decreasing filtration in contralateral kidneys (17, 18). Paradoxically, analysis of split renal function in this patient showed no improvement of sGFR in the ischemic kidney but a slight reduction of sGFR in the contralateral kidney. Although we believe that the result of preservation of the ischemic kidney function without further deterioration is acceptable, it is contradictory to the long-term follow-up outcome that presents a reduction in serum creatinine and an increase in overall eGFR. Renal dynamic scintigraphy is viewed as an effective tool in assessing the change in sGFR in the ischemic kidney (16, 17). However, measurement bias of renal dynamic imaging at different time points by different nuclear medicine physicians should be taken into consideration. We recommend the use of the right-to-left sGFR ratio as a potential solution. Although GFR of the ischemic kidney in this patient remained stable after stenting, its proportion in total renal function increased. This result probably reflected the recovery of the ischemic kidney, on the other hand. However, the efficiency of this index needs to be tested in larger trials.

So, how do we recognize patients for whom renal function can be salvaged? Preserved blood flow and kidney structure might be associated with renal outcomes after recanalization. ACC/AHA guidelines suggested that patients with an atrophic kidney size of pole-to-pole length <7 cm are unlikely to benefit from revascularization (9). However, recent studies demonstrated that patients might benefit from recanalization when collateral flow preserves even if the atrophic kidney is smaller than the indicated size in the guidelines (11, 12). Organ viability could be preserved with complete arterial occlusion in the presence of collateral arterial supply (19). Metabolic needs of the kidney only require 10% of the oxygen supplied from arterial blood (20). Collateral circulation is capable of protecting the ischemic kidney from infarction when the main renal artery is occlusive (12). There is mounting evidence indicating that for RAO patients, angioplasty may be clinically feasible in the kidney with a preserved subsegmental collateral perfusion.

Being considered as a noninvasive, side-effect-free, and cost-effective method, Doppler ultrasound has become routine and the first-line clinical examination in the diagnosis of RAS. Evaluation by ultrasound is mostly based on direct hemodynamic parameters including peak systolic velocity and renal aortic ratio and indirect parameters such as resistance index and acceleration time (21, 22). These criteria are affected by lesions in the kidney and other systemic factors of patients (22). Although the reported sensitivity and specificity of Doppler ultrasound can both reach more than 95% by the hands of experienced sonographer (23), assessments are not made on the basis of changes in vascular luminal diameter. The above-mentioned limitations of Doppler ultrasound can partly explain the inconsistency of results between ultrasound and angiography in this patient.

In summary, this case demonstrated that revascularization is feasible in RAO patients who have preserved collateral flows after optimal medical therapy fails. Even if RAO patients do not have refractory hypertension or fit any clinical trial inclusion criteria by far. Moreover, we found that the results of sGFR measured by renal dynamic scintigraphy might not be in agreement with the total eGFR calculated by the MDRD formula. The right-to-left sGFR ratio could be considered a new index when evaluating the efficacy of angioplasty in RAS patients.

## Data Availability

The original contributions presented in the study are included in the article/supplementary materials; further inquiries can be directed to the corresponding author.
